# Study of BBB Dysregulation in Neuropathogenicity Using Integrative Human Model of Blood–Brain Barrier

**DOI:** 10.3389/fncel.2022.863836

**Published:** 2022-06-10

**Authors:** Coraly Simöes Da Gama, Mélanie Morin-Brureau

**Affiliations:** Inserm, Sorbonne University, UMRS 938 Saint-Antoine Research Center, Immune System and Neuroinflammation Laboratory, Hôpital Saint-Antoine, Paris, France

**Keywords:** *in vitro* model, human, blood-brain barrier, 3D models, transwell

## Abstract

The blood–brain barrier (BBB) is a cellular and physical barrier with a crucial role in homeostasis of the brain extracellular environment. It controls the imports of nutrients to the brain and exports toxins and pathogens. Dysregulation of the blood–brain barrier increases permeability and contributes to pathologies, including Alzheimer's disease, epilepsy, and ischemia. It remains unclear how a dysregulated BBB contributes to these different syndromes. Initial studies on the role of the BBB in neurological disorders and also techniques to permit the entry of therapeutic molecules were made in animals. This review examines progress in the use of human models of the BBB, more relevant to human neurological disorders. In recent years, the functionality and complexity of *in vitro* BBB models have increased. Initial efforts consisted of static transwell cultures of brain endothelial cells. Human cell models based on microfluidics or organoids derived from human-derived induced pluripotent stem cells have become more realistic and perform better. We consider the architecture of different model generations as well as the cell types used in their fabrication. Finally, we discuss optimal models to study neurodegenerative diseases, brain glioma, epilepsies, transmigration of peripheral immune cells, and brain entry of neurotrophic viruses and metastatic cancer cells.

## Introduction

**Figure 1:**
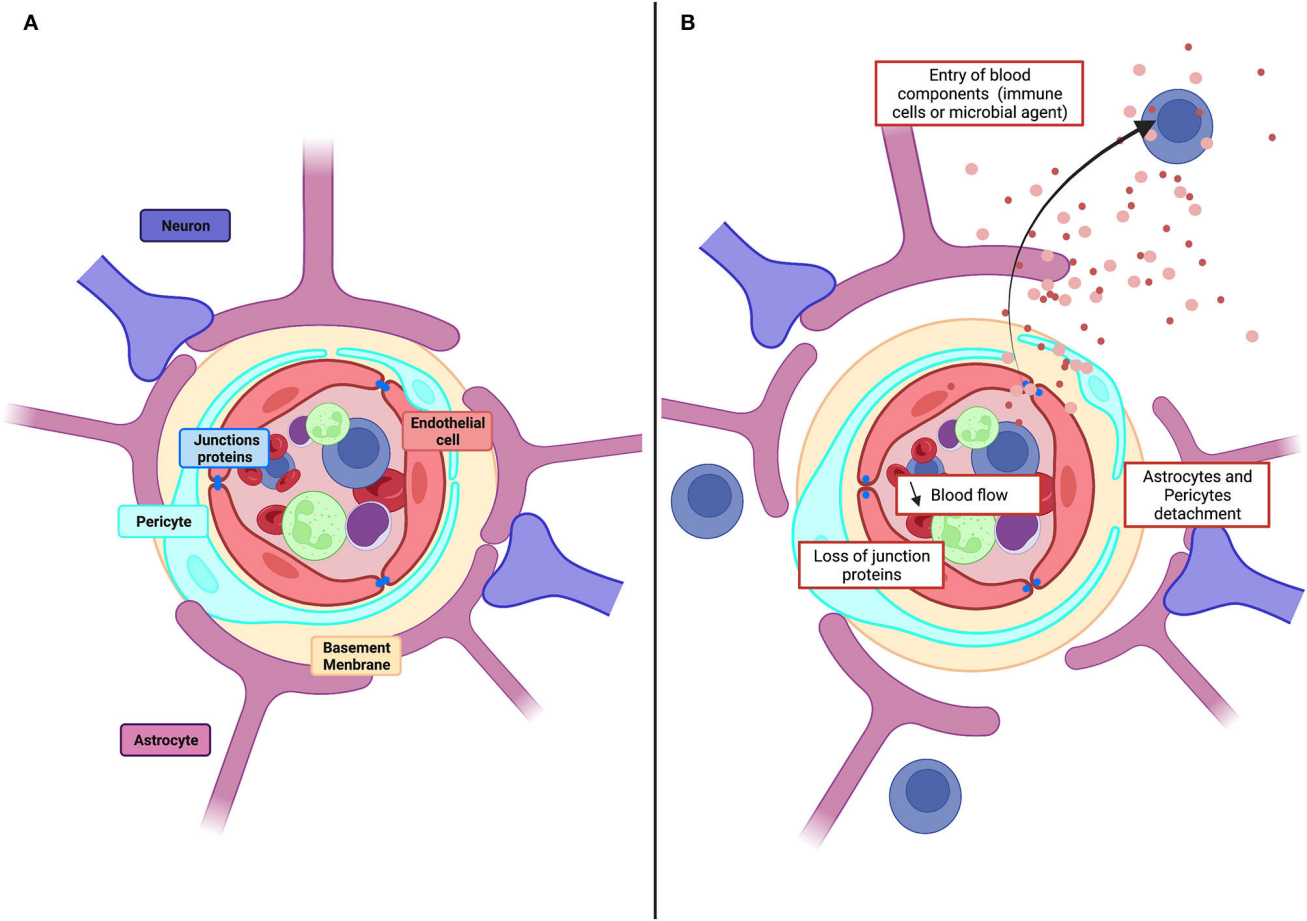
Physiological structure of the BBB and pathological alteration. **(A)** Schematic illustration of cellular constituents of BBB: the anatomical seat of the BBB is endothelial cells, associated and supported by pericytes and astrocytic end-feet forming the cellular barrier. Endothelial cells are connected to each other by tight junction proteins which ensure the impermeability of the barrier and form a physical barrier. **(B)** In pathological conditions, the BBB will be affected, and there will be a decrease in blood flow, detachment of pericytes and astrocytes as well as loss of tight junction proteins. All this giving the possibility for toxic molecules to infiltrate the brain parenchyma.

The blood–brain barrier (BBB) controls molecular and ionic fluxes at brain capillaries and maintains the extracellular neuronal environment to ensure proper central nervous system function. The BBB is a functional assembly of brain endothelial cells (BECs), pericytes, and astrocytes (Figure 1) (Segarra et al., 2019). BECs express tight junction (TJ) proteins and transporters. They are characterized by few caveolae and many mitochondria and generate a high transendothelial electrical resistance (TEER) and low paracellular fluxes. Pericytes and astrocytes are involved in endothelial cell proliferation and polarization and in TJ formation and maintenance (Gökçinar-Yagci et al., 2015; Guérit et al., 2021). Intercellular communication influences transcript expression resulting in a BBB which adapts continuously to changes, including aging, development, and nutrition (Segarra et al., 2021).

Dysregulations of the BBB reduce blood flow and permit entry to the brain of toxic molecules, immune cells, and microbial agents (Kisler et al., 2017). Vascular damage is evident as a loss of tight junction proteins on endothelial cells, an increase of permeability and the detachment of astrocytes and pericytes (Figure 1) Dysregulation contributes to cerebrovascular accidents, generates inflammation, and is linked to neurodegenerative diseases, epilepsies, and neuroinflammatory disorders, including multiple sclerosis (Engelhardt and Ransohoff, 2005; Yang and Rosenberg, 2011; Sweeney et al., 2019). Vascular disruption is associated with cognitive decline in degenerative diseases, including Alzheimer's disease (AD) (Zlokovic, 2011), Parkinson's disease (PD) (Malek et al., 2016), Huntington's disease (HD) (Lin et al., 2013), and amyotrophic lateral sclerosis (ALS) (Winkler et al., 2013). Markers of vascular damage typically appear before neurodegeneration and cognitive impairment become evident. In the epilepsies, BBB function is degraded further by seizures as the disorder progresses: TJs (Rigau et al., 2007) and pericytes are lost (Milesi et al., 2014), efflux pumps, including the P-glycoprotein pump (P-gp), are degraded (Gil-Martins et al., 2020), and extravasation of blood components is facilitated (van Vliet et al., 2007; Michalak et al., 2012). BBB breakdown is an initial insult in multiple neurological disorders. Neuronal damage is aggravated by the entry of blood proteins to the brain, by the loss of intracerebral homeostasis due to transporter dysregulation, and by the neuroinflammation induced when peripheral immune cells enter the brain (Sweeney et al., 2019).

The first model studies on the BBB were made in rodents *in vivo*. Their advantages were that BBB architecture was not perturbed and that blood cell movements in capillaries generated shear stress, crucial for BBB maintenance. Disadvantages included high cost, slow time course, and ethical concerns. Notably, molecules with useful effects on the BBB in studies *in vivo* did not always translate successfully in clinical trials. Even though structural elements of the BBB are similar, there are molecular differences between animals and humans (O'Brown et al., 2018). Transcriptomic analysis of mouse and human brain vessels has revealed distinct patterns of gene expression, including those coding for key tight junction proteins and some transporters (Urich et al., 2012; Hoshi et al., 2013; Song et al., 2020). Brain imaging and modeling work point to the differences in activity of the P-glycoprotein (P-gp) transporter (Syvänen et al., 2009; Verscheijden et al., 2021). Aquaporin-4 (AQP4) is less strongly expressed in human astrocytic end-feet (Eidsvaag et al., 2017). Astrocytes of human cortex are larger and more complex than those in mice (Oberheim et al., 2009) and may respond differently in neurodegenerative conditions (Colombo et al., 2002). These differences have been an incentive to develop human models of the BBB.

*In vitro* models of the BBB based on human cells have several advantages: (i) cellular environment may be easily and reproducibly controlled; (ii) improved accessibility permits mechanistic analysis; (iii) ethical concerns are reduced; and (iv) personalized medicine for specific patients may be possible. These models were first used to examine strategies to decrease BBB-dependent pharmacoresistance in neuropathologies. The use of cells from patients has permitted attempts to reproduce BBB structure and function in specific disease states and move toward personalized therapies. Human models facilitate work on BBB degradation due to extrinsic factors, such as viral infection or brain trauma, or to diseases, such as diabetes and hypertension. Progress is still necessary to define the mechanisms of BBB dysfunction and their role in disease progression. Many reviews have described how *in vitro* models have examined BBB permeability to drugs, but models also permit advances in elucidating pathological mechanisms. This review traces how advances in understanding of BBB cell types and their interactions have improved models that examine these questions in human neurological diseases.

## Modeling Human BBB *in vitro*

**Figure 2:**
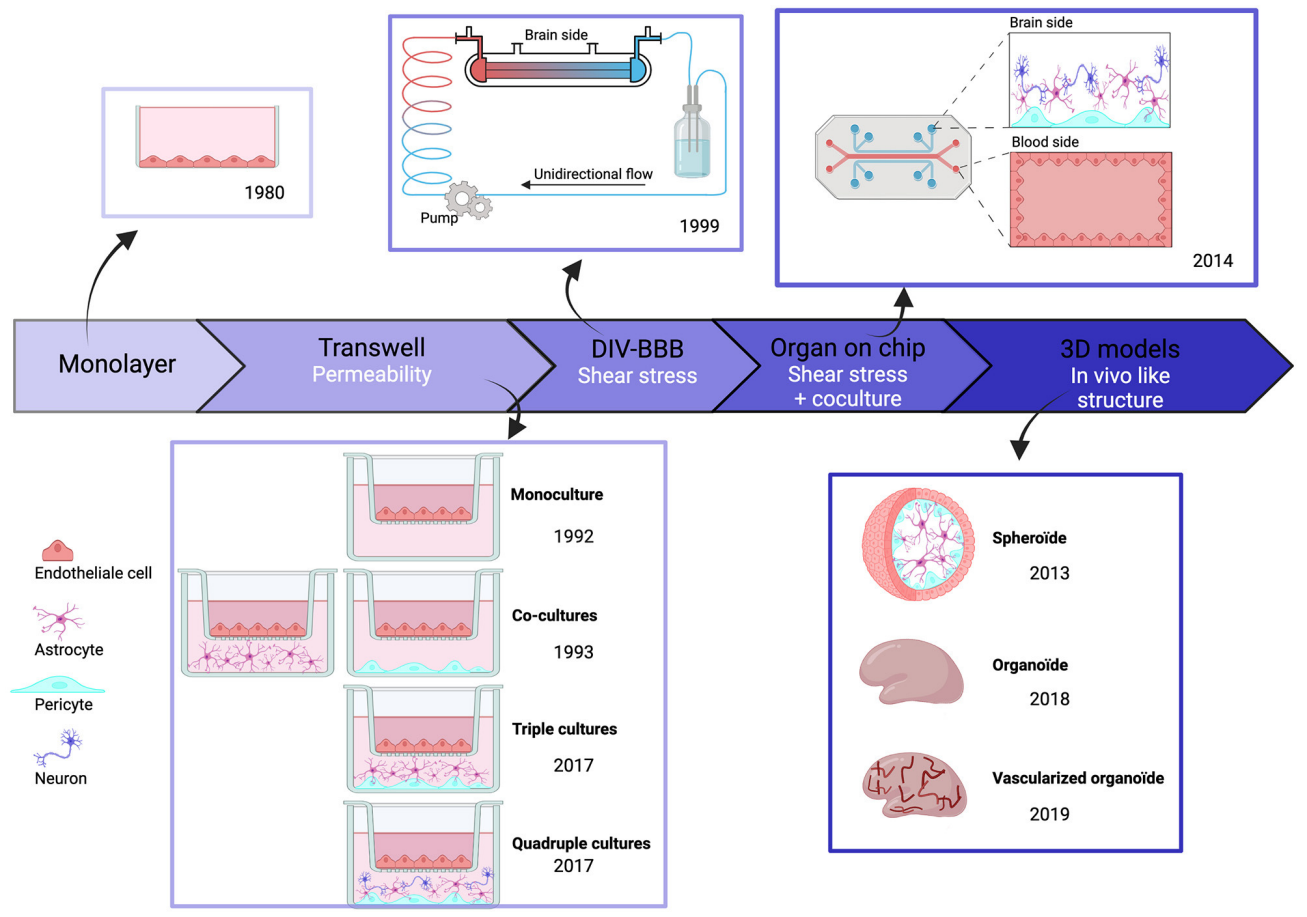
Chronologic event in human BBB invitro models. Overtime, BBB models have evolved and became more complex to best reproduce physiological conditions. But each of these models have themselves evolved, in particular through the evolution of cell types.

The first *in vitro* BBB models, published in the 1980s, consisted of cultured monolayers of rodent endothelial cells. An *in vitro* model based on human brain endothelial cells (hBECs) first appeared in 1985 (Pardridge et al., 1985). We examine here how these models have been improved with attention to physiological dimensions, geometry, and proximity to different cell types and simulation of blood flow past exposed endothelial cells (Figure 2).

The improvement in measured values for BBB permeability was an initial focus in work on transwell systems. Monolayers of hBECs, cultured on semipermeable polystyrene or polycarbonate membranes, were used to examine the fluxes of fluorescent tracers and immune cells between separate compartments (Dehouck et al., 1992). Whereas initial models only used hBECs, it became clear that both astrocytes and pericytes are essential to maintain and develop BBB properties (Gökçinar-Yagci et al., 2015; Guérit et al., 2021). This problem was resolved by transwell models comprising co-cultures with other cell types (Hurwitz et al., 1993). Typically, hBECs were grown in the upper transwell compartment, and astrocytes and pericytes on the lower side of the membrane, although further work suggests that this separation may not be ideal (Kulczar et al., 2017). Neurons are suggested to affect BBB properties *in vivo* (Li et al., 2014) and they influence BBB properties when present in transwell cultures (Stone et al., 2019). Overall, transwell models are easy to setup, reproducible, and scalable. They permit molecular studies using immunohistochemistry or proteomic and genomic techniques on cells detached from membranes. However, changes in two factors improved human BBB model performance. Cell–cell contacts in 3D among hBECs, astrocytes, pericytes, and neurons were poorly reproduced in the transwell configuration. Furthermore, the absence of blood flow and the resulting shear stress altered the properties of endothelial cells in transwell models (Cucullo et al., 2011).

The first BBB model to include potential effects of shear stress and 3D structure was based on an artificial capillary formed by a polycarbonate fiber pre-coated with fibronectin, rat collagen, or poly-D-lysine. In this dynamic *in vitro* BBB (DIV-BBB) model, published in 1999, endothelial cells were seeded on the intra-luminal side with astrocytes and pericytes on the extraluminal side. Cells communicated *via* 0.4-um pores in the hollow fiber. The flow was generated by a pump reproducing pulsatile pressure patterns (Cucullo et al., 2002) to induce shear stress, crucial for BBB maintenance (Cucullo et al., 2011). BBB parameters improved to the levels close to those measured *in vivo*, including a low permeability, TEER values 10 times higher than in transwells, and an enhanced expression of pump molecules. However, the fiber diameter was larger than physiological values and the model did not explicitly distinguish between venules and capillaries. These concerns were later addressed by using separate fibers for capillaries and venules and replacing astrocytes with human smooth muscle cells (Cucullo et al., 2011).

Even with these advantages, DIV-BBB models were superseded by BBB-on-chip models based on nano-fabrication and microfluidic techniques (Ma et al., 2005). In the earliest iterations, monolayer endothelial cells alone (BBB-on-chip) or with astrocytes and pericytes (NVU-on-chip) were cultured as in transwell systems (Jiang et al., 2019). Most models used sandwich structures, as previously, to separate neural and vascular chambers, and microfluidic channels were formed from biocompatible organic polymers. Three categories of chip material have been used. (i) natural materials, such as collagen or gelatin, similar to extracellular matrix; (ii) synthetic materials, including biodegradable polylactic acid (PLA), polyglycolic acid (PGA), and its copolymer PLGA or polydimethylsiloxane (PDMS); and (iii) hybrid natural-synthetic materials with advantages of both materials. They take into account the biocompatibility of natural components, cell adherence, cell response, and immune reaction as well as the physicochemical proprieties of synthetic components to increase mechanical strength (Reddy et al., 2021). For example, PDMS is optically transparent, so microscopy is possible and is permeable to gas and water for cell culture. Porous membranes provide a platform for co-culture and permit exchange between luminal and abluminal sides (Jiang et al., 2019). Several configurations have been developed. In the SyM-BBB system, trapezoidal structures were used to model blood and brain compartments connected with 3-μm microgaps (Prabhakarpandian et al., 2013). Attempts were made to reproduce the circular cross-section of blood vessels using a 3D printing frame coated with collagen (Kim et al., 2015). However, the diameter of artificial vessels (100 μm) was greater than that of brain capillaries and venules (10–90 μm) (DeStefano et al., 2018). More recent BBBs on chip models have used a vasculogenesis strategy attempting to reconstruct vasculature *de novo* by inserting cells into Matrigel or self-polymerizing extracellular matrix protein (ECM). The resulting BBB-on-chips are anatomically similar to brain capillaries since endothelial cells self-assemble into a vascular network with pericytes and astrocytes at their surface (Campisi et al., 2018). However, work is still needed to improve reproducibility due to heterogeneity in branching patterns.

This generation of BBB models has exploited 3D printing and bioprinting (Ho et al., 2015), reducing the time and technical expertise needed to reproduce complex model architectures. Interconnectivity, size, and geometry of model components and cell types can be controlled (Norman et al., 2017). The use of 3D printing techniques in models is growing in maturity.

Organoid BBB models emerged together with the growth of BBB-on-chip models. They consist of spheroid *in vitro* cell assemblies which often closely resemble *in vivo* structures (Pacitti et al., 2019). The first BBB organoids consisted of human endothelial cells, astrocytes, and pericytes, which self-assemble under low-adherence conditions (Urich et al., 2013). These three cell types contact each other directly, enhancing the relevance of BBB organoids, even if microglia and neurons, which also affect the BBB, are absent. Lancaster et al. (2013) have grown 3D whole-brain organoids from iPSCs obtained by reprogramming fibroblasts on stem cells and then differentiated using a chemically defined medium. They can be maintained over a year, but tend to necrosis in central parts of the structures. Efforts have been made to induce vascularized human brain organoids. Matrigel coating of organoids with iPSC-derived ECs from the same individual reportedly results in a vascularized brain organoid (Pham et al., 2018). Recent efforts to produce a 3D BBB model have used six cortical cell types: (i) human brain microvascular endothelial cells (HBMEC); (ii) human pericytes (HBVP); (iii) human astrocytes (HA); (iv) human microglia (HM); (v) human oligodendrocytes (HOs); and (vi) human neurons (HN). This model exhibits functional responses to hypoxia and neurotoxicity (Nzou et al., 2018). More comprehensive testing and validation of organoid BBB models need to be done, but these models seem likely to be very useful for work on neuropathologies.

Organotypic cultures of brain tissue have been used to study neuronal activity and cellular interactions since the 1970s. They have the advantage that spatial relations between cell types are maintained. The finding that blood vessels of organotypic culture respond to angiogenic stimuli led to their use as a BBB model (Moser et al., 2003; Hutter-Schmid et al., 2015). Capillary endothelial cells express tight junction proteins and transporter molecules, including P-gp and GLUT1 (Camenzind et al., 2010; Morin-Brureau et al., 2013). Co-cultures of organotypic brain tissue overlaid on endothelial cells have permitted microdialysis experiments and measurements of TEER (Duport et al., 1998; Zehendner et al., 2014). Most organotypic culture work has been done on rodent tissue. However, organotypic cultures of human tissue obtained after surgery on epileptic patients have been used to study neuronal and seizure activity, and BBB studies could clearly be envisaged (Le Duigou et al., 2018).

## Which Cell Types Are Optimal to Model BBB?

### Cell Lines and Primary Cells

Most human cell lines or primary cell lines used in BBB models are available commercially. Primary cells of the NVU may also be isolated in the laboratory (Bernas et al., 2010). However, since it may be difficult to obtain human tissue for reasons, including ethics, many models have been based on the immortalized cell line hCMEC/D3. These cells were derived from brain microvessels isolated from the human temporal lobe. hBECs were sequentially immortalized by lentiviral vector transduction *via* expression of the catalytic subunit of human telomerase (hTERT) and SV40 large T-antigen (Weksler et al., 2005). The properties of this hBEC cell line have been characterized in more than 400 publications. Other cell lines may have advantages. Human brain microvascular endothelial cells (HBMECs) were isolated from the human brain and immortalized by simian virus 40 large T-antigen (SV40-LT) (Stins et al., 2001). The use of HBMEC cells in BBB models was validated by their permeability to compounds, such as caffeine (Eigenmann et al., 2016). The immortalized cell line BB19 was derived from human brain endothelium transformed with E6E7 genes of the human papilloma virus. These cells form tubules in Matrigel and express appropriate tight junction and transporter protein markers (Kusch-Poddar et al., 2005). Another group of cell lines TY08/TY10 was based on BECs isolated from a patient with meningiomas and immortalized with a temperature-sensitive SV40 large T-antigen (Sano et al., 2010).

BB19 and TY10 cell lines retain most anatomical and functional characteristics of BECs. In comparison with the hCMEC/D3 and hBMEC lines, expression of the tight junction molecule ZO-1 is reduced. In contrast, claudin-5 and VE-cadherin are expressed at higher levels in the TY10 than in the hBMEC cell line. BB19 cells express only low levels of junctional proteins (Eigenmann et al., 2013). Performance of the hCMEC/D3 and immortalized human brain endothelial cell lines (HBMEC/ciβ), measured with TEER and expression of tight junction proteins, was enhanced by compounds, such as hydrocortisone or lithium (Furihata et al., 2015; Laksitorini et al., 2019). Proteomics suggests that hCMEC/D3 lines have enhanced efflux transport whereas HBMEC/ciβ have more IgG-transport activity (Masuda et al., 2019). hCMEC/D3 cultures conserve immune profiles and permit the transmigration of immune cells (Daniels et al., 2013). Co-culture with astrocyte and pericyte cell lines does not change TJ expression or TEER values indicating the importance of the choice of the model. A tri-culture model has been developed by culturing HBMEC cells with immortalized human astrocyte (HASTR/ci35) and pericyte cells (HBPC/ci37) (Ito et al., 2019). Cryo-conservation methods have been improved to preserve mono- and co-cultures (Marquez-Curtis et al., 2021).

Blood–brain barrier models based on these immortalized or primary human cells have suffered from constraints in obtaining human tissue. Furthermore, the human hBEC phenotype is not always well maintained in culture and TEER values remain lower than data from rodents *in vivo*. *In vitro* human BBB models have therefore explored stem cells.

### Human Pluripotent Stem Cells (hPSCs), Human Embryonic Stem Cells (ESCs), and Induced Pluripotent Stem Cells (iPSCs)

As human iPSC technology has advanced, protocols have been developed to generate brain endothelial cells, distinct from peripheral endothelial cells. The most frequently used protocol, established by Lippmann et al., involves co-differentiation of endothelial cells with neural cells followed by endothelial cell purification (Lippmann et al., 2012). This protocol has since been adapted with direct differentiation of iPSC to brain endothelial cells using a chemically defined medium (Qian et al., 2017). Several adaptations have been made to enhance barrier properties. For example, the presence of retinoic acid during differentiation increases VE-cadherin expression and TEER (Lippmann et al., 2014). Endothelial cell differentiation can be accelerated by optimizing the culture medium or seeding (Wilson et al., 2015; Hollmann et al., 2017), by co-culture with astrocyte iPSCs (Neal et al., 2019), and by protecting against damage during freeze-thaw cycles (Yamashita et al., 2020). Transcription factor supplements also increase the resistance and maintenance of BBB models based on iPSCs (Roudnicky et al., 2020). Even so, transcriptomic analyses have revealed the presence of epithelial markers, such as claudin-7, raising questions about the identity of differentiated cells (Delsing et al., 2018). Several other promising protocols exist. Praça et al. (2019) used iPSCs generated from human cord blood cells or fibroblasts and defined medium with several growth factors to obtain cells that express TJs, endothelial markers, and metabolic transporters. Nishihara et al. (2020) developed a method for human induced pluripotent stem cells based on erythrocyte reprogramming with specific transcriptional factors (oct4, shRNA-p53, SOX2, KLF-4, L-Myc, and Lin28) and then differentiated using chemically defined medium. Overall, we note that barrier properties of iPSC-derived culture models based on the Lippman method are comparable to data from *in vivo* work, despite the debate on epithelial cell markers.

Pericytes have been derived with iPSCs protocols from neural crest stem cells (NCSCs). After adding bFGF and EGF to culture medium, mural cells expressed aSMA and SMA-22 but not NG2+ and PDGFRβ (Wang et al., 2012). Transcriptomic analysis of brain mural cells *in vivo* shows low expression of SMA but high expression of NG2+ and PDGFRβ (Vanlandewijck et al., 2018). Stebbins et al. (2019) using a medium with added fetal bovine serum showed that NCSCs were induced into pericytes expressing NG2, PDGFRβ, and αSMA^low^ (Gastfriend et al., 2021). Alternatively, pericytes have been induced from mesodermal intermediates. iPSC-derived pericytes from either mesodermal or NCSCs exhibit similar pericyte markers and barrier properties (Faal et al., 2019).

Several protocols have been used to derive astrocytes from iPSCs. However, long-term co-cultures with neural stem cells have proven difficult due to the relative immaturity of astrocytes (Krencik et al., 2011). Development may be accelerated by remodeling chromatin and overexpressing gliogenic FT NF1A and SOX9 (Krencik et al., 2011; Majumder et al., 2013). Even so, differentiation of astrocytes expressing GFAP, CD44, and S100b or EEAT1/2 occurs slowly over a month.

A co-culture model of iPSC-derived microglia and iPSC-derived endothelial cells has recently been developed. iPSC-derived microglia were obtained first from homogeneous and stable embryonic bodies (EB), and after 30 days, macrophage precursors were differentiated into microglia in the presence of IL-34, M-CSF, and TGFβ-1 (Reich et al., 2020). Media with no serum enabled stable co-cultures of iPSC-derived microglia and brain endothelial cells which respond appropriately to inflammatory stimuli, such as lipopolysaccharide (Bull et al., 2022).

### Induced Pluripotent Stem Cells

Induced pluripotent stem cells have been used in multiple configurations: static transwell mono- or co-cultures (Stebbins et al., 2019) and monoculture microfluidic models (Faley et al., 2019), co-culture with cell lines (Park et al., 2019) or with iPSC-derived neuron or pericyte (Jamieson et al., 2019; Vatine et al., 2019). Induction of shear stress by exposure to fluid movement decreased apoptosis, enhanced proliferation, and the mobility of endothelial cells in these models but did not alter the expression of TJ proteins or P-gp (DeStefano et al., 2018). TEER values approached *in vivo* levels after ~12 days, and integrity is maintained over 3 weeks in culture.

Human iPSC approaches permit studies on cells derived from patients (Wilson et al., 2015) to identify disease mechanisms (Lim et al., 2017) and signaling pathways (Vatine et al., 2017). Further development of iPSC techniques should facilitate this approach. Cerebrovascular damage may result from the genetic background but also from environmental, vascular, or lifestyle-related risk factors. In Alzheimer's disease (AD), pericyte degeneration, for instance, is more likely in patients who carry the ε4 allele of apolipoprotein E (APOE^*^ε4) (Halliday et al., 2016). Using iPSC-derived EC from Qian's et al.'s method, several discoveries on genetic impact have been made in AD. Transwell BBB models with iPSC-derived endothelial cells from AD patients with a PSEN-1 mutation have found distinct BBB properties, including responses to ultrasound, used to permit drug delivery (Oikari et al., 2020). iPSC-based models have demonstrated a potential mechanism for the genetic susceptibility of APOE4 for cerebral amyloid angiopathy (CAA) associated with Alzheimer's disease (Blanchard et al., 2020). iPSCs derived from patients with ALS due to C9orf72 mutation have shown differences in regulation of the P-gp (Mohamed et al., 2019). In this study, iPSCs have been differentiated using DMEM/F12 medium supplemented with L-glutamin, heparin, and B27 for astrocytes or vasculife basal medium supplemented with FGF, acid ascorbic, hydrocortisone, glutamine, and growth factor for brain endothelial cells. A comparison of iPSC-derived BECs from control and patients with Huntington's disease (HD), differentiated using Lippman's method, revealed HD-related defects in angiogenesis and BBB maturation related to WNT pathway dysregulation (Lim et al., 2017).

## Validation of *in vitro* Human BBB Models and Specific Adaptation for Studies on BBB Neuropathogenicity

How should BBB models be validated? Can specific BBB models be best used to study distinct pathologies? For validation, we examine resistance measurements and marker molecule expression. As neurological pathologies, we examine gliomas, neurodegenerative diseases, and epilepsy which impose different constraints.

### Permeability of the Barrier

Several methods have been used to evaluate BBB permeability. The transendothelial electrical resistance (TEER) is a quantitative marker of BBB integrity and is evaluated by Ohm's law or impedance spectroscopy (DeStefano et al., 2018). TEER has been measured in rodent BBB *in vivo* as 5,000 ohm/cm^2^ with physiological values that may be as low as 1,500 ohm/cm^2^. Transendothelial electrical resistance values higher than 500 ohm/cm^2^ measured from *in vitro* models are considered to reflect an intact BBB (Mantle et al., 2016). Published TEER values vary greatly according to cell type, culture model, and the measurement technique (Srinivasan et al., 2015). TEER values in models based on hCMEC/D3 cells co-cultured with astrocytes increased to 140 ohm/cm^2^ when measured with epithelial volt/ohm meter (EVOM) techniques (Daniels et al., 2013). Values up to 2,940 ohm/cm^2^ have been measured from iPSC-derived BMECs treated with retinoic acid and as high as 4,000 ohm/cm^2^ for tri-cultures of human pericytes, astrocytes, and neurons derived from progenitor cells (Lippmann et al., 2014). Larger pore sizes of the membrane in transwell models permit cell migration and allow contact between astrocytes and endothelial cells *via* end-feet astrocytes (Niego and Medcalf, 2013). However, pore size is known to affect TEER values in mouse models (Wuest et al., 2013). In several human cell lines, pore size increases are correlated with higher TEER values measured with EVOM (Eigenmann et al., 2013). Several other factors, including temperature, pore density, and cell insert properties, have been shown to influence TEER values (Vigh et al., 2021). Culturing hiPSC-derived brain endothelial cells on laminin-511 increases and stabilizes TEER over 15 days (Motallebnejad and Azarin, 2020).

Functional BBB permeability has also been evaluated by measuring the passage of fluorescent molecules of various sizes. The most frequently used tracers are lucifer yellow (LY: 444 Da), fluorescein sodium (NaF: 376 Da) or sucrose (342 Da), and fluorescent dextran (70 Kda) for larger molecules. Cell lines and culture conditions influence permeability. hCMEC/D3 cultures are permeable to small molecules, such as sucrose or mannitol (25.10^−6^ cm/s), whereas models based on human pluripotent stem cells are impermeable (0,6.10^−6^ cm/s) (Eigenmann et al., 2013). Measuring TEER and movements of fluorescent markers are complementary techniques for the evaluation of barrier permeability.

### Junctional Protein, Adhesion Molecules, and Transporters

Verifying the expression of relevant cell markers is another key element of model validation. The most common endothelial cell markers used are VE-cadherin, PECAM-1 or VWF, ZO-1, claudin-5, and occludin which are clustered at tight junctions whereas GLUT1 labels transporters and P-gp drugs efflux sites (DeStefano et al., 2018). Transcriptomics has been used to evaluate gene expression and western blot analysis to measure protein levels, with immunofluorescent staining needed to verify a correct subcellular protein localization. Transporter function has been measured using drug molecules or fluorescent proteins, such as rhodamine123. hCMEC/D3 is the most characterized cell line used in BBB models. Transcriptomic analysis has revealed 144 SLC transporters and 23 ATP-binding cassette (ABC) efflux transporters (Carl et al., 2010). Rhodamine-3 accumulation assay has shown that this cell line expresses a functional P-gp (Tai et al., 2009). mRNA expression of transporters and junctional protein localization at membrane sites in iPSC-derived endothelial cells are further useful indices (Delsing et al., 2018). While most cell lines express adherens junction (AJ) and TJ, localization and density are influenced by culture conditions, including cell confluence. Supplementing culture medium with hydrocortisone or lithium chloride (LiCl) improves TJ and AJ expression. Indeed, LiCl activates the Wnt/β-catenin signaling pathway, which controls the expression of claudins (Liebner et al., 2008). Growth factors also affect tight junction formation: basic fibroblast growth factor (bFGF) enhances TJ density, whereas it is reduced by the vascular endothelial growth factor vascular endothelial growth factor (VEGF) (Morin-Brureau et al., 2011). In iPSC-based models, the transcription factors, such as SOX18, TLAL1, SOX7, and ETS1, act in synergy to enhance BBB function and integrity (Roudnicky et al., 2020).

Immune cell trafficking across the BBB is a higher-level function that has been tested by examining the expression of adhesion molecules, including ICAM1, VCAM1, and PECAM1. The expression of these molecules has been shown to be enhanced by the proinflammatory cytokines, such as TNF-α and IFN-γ (Nishihara et al., 2021). In contrast, exposure to TNF-α, IL-8, and IL1-β decreased barrier properties (Vatine et al., 2019).

### Validation of BBB Models for Glioma

Blood vessels inside a brain tumor differ from those surrounding the tumor and those at a distance. Inside a tumor, vessel fenestrations are much increased and permeability varies widely. These distinct properties have been recognized as a brain–blood tumor barrier (BBTB) (Arvanitis et al., 2020). The BBTB exhibits that an enhanced angiogenesis (Jain et al., 2007) increased inflammation (Sowers et al., 2014) and a lower blood flow due to tumor growth. The leakiness of blood vessels inside the tumor is heterogenous (Seano et al., 2019). The properties of BBTB in low-grade glioma are closer to those of the BBB. Models of BBTB have used primary glioma cells to reproduce tumor environment (van Tellingen et al., 2015). Transwell models have been adapted to study metastatic migration across the BBB when cerebral tumor is a metastatic brain tumor in consequence of invasion of breast cancer cells(Vandenhaute et al., 2016).

### Validation of BBB Models for Neurodegenerative Syndromes

The role of the BBB in neurodegenerative diseases is most usefully examined with models that include neurons. They should, for instance, reproduce elevated levels of cerebral proteins in the extracellular matrix (ECM) and also permit studies on diseases with genetic and/or sporadic elements. Neurodegenerative disease may be sporadic, or familiar with a genetic component. One familial risk factor is the allele of apolipoprotein E (APOE^*^ε4) associated with Alzheimer's disease (AD) (Saunders et al., 1993). iPSC-derived cells permit the exploration of genetic factors in BBB models. Monolayers of hiPSC-derived endothelial cells from Alzheimer's patients with PSEN1 and PSEN2 mutations, differentiated with a chemically defined medium, possessed a low TEER, reduced expression of claudin-5, occludin, and ZO1 protein, and impaired glucose transport (Raut et al., 2021). A 3D microfluidic BBB model is based on iPSCs from patients with AD mimicked vascular damage as an increased BBB permeability, a reduction in TJs and AJs, and an increased expression of the matrix-metalloproteinase-2 (MMP2) (Shin et al., 2019).

Blood–brain barrier disruption in PD has been modeled with a human BBB-on-chip cultivated under flow conditions and containing iPSC-derived dopaminergic neurons, astrocytes, microglia, and pericytes differentiated with cell-specific chemically defined medium and hBECs using Qian's method from patients. BBB function was degraded in this model which reproduced the aspects of PD, including alpha-synuclein accumulation and mitochondrial impairment (Pediaditakis et al., 2021).

Blood–brain barrier models have also used iPSCs, differentiated by Qian's method, derived from ALS patients with SOD4 or C9orf72 mutations. BBB dysfunction was revealed as a reduced TEER and an upregulated P-gp transporter (Qosa et al., 2016; Katt et al., 2019).

### Validation of BBB Models for Epilepsies

Vascular damage is a key factor in epileptogenesis since it permits extravasation of peripheral immune cells from the circulation which augment inflammatory responses and increase neuronal excitability (Löscher, 2020). Excessive neuronal firing during a seizure initiates BBB degradation. A human model has been developed by co-culture of neurons with HBMEC cells, astrocytes, and pericytes, but has not yet been used to study the epilepsies (Stone et al., 2019). Some mechanisms have been studied in rodent organotypic cultures. In 1998, Duport et al. (1998) developed a co-culture system of endothelial cells and organotypic cultures. Work on rodent organotypic cultures has shown that neuronal activity induces VEGF-A secretion, which initiates angiogenesis and downregulates ZO-1 (Morin-Brureau et al., 2011). Human organotypic cultures of tissue from epileptic patients could be used to examine the effects of seizure-like activity on BBB properties (Le Duigou et al., 2018).

## Impact of Inflammatory Processes on BBB Integrity

**Figure 3:**
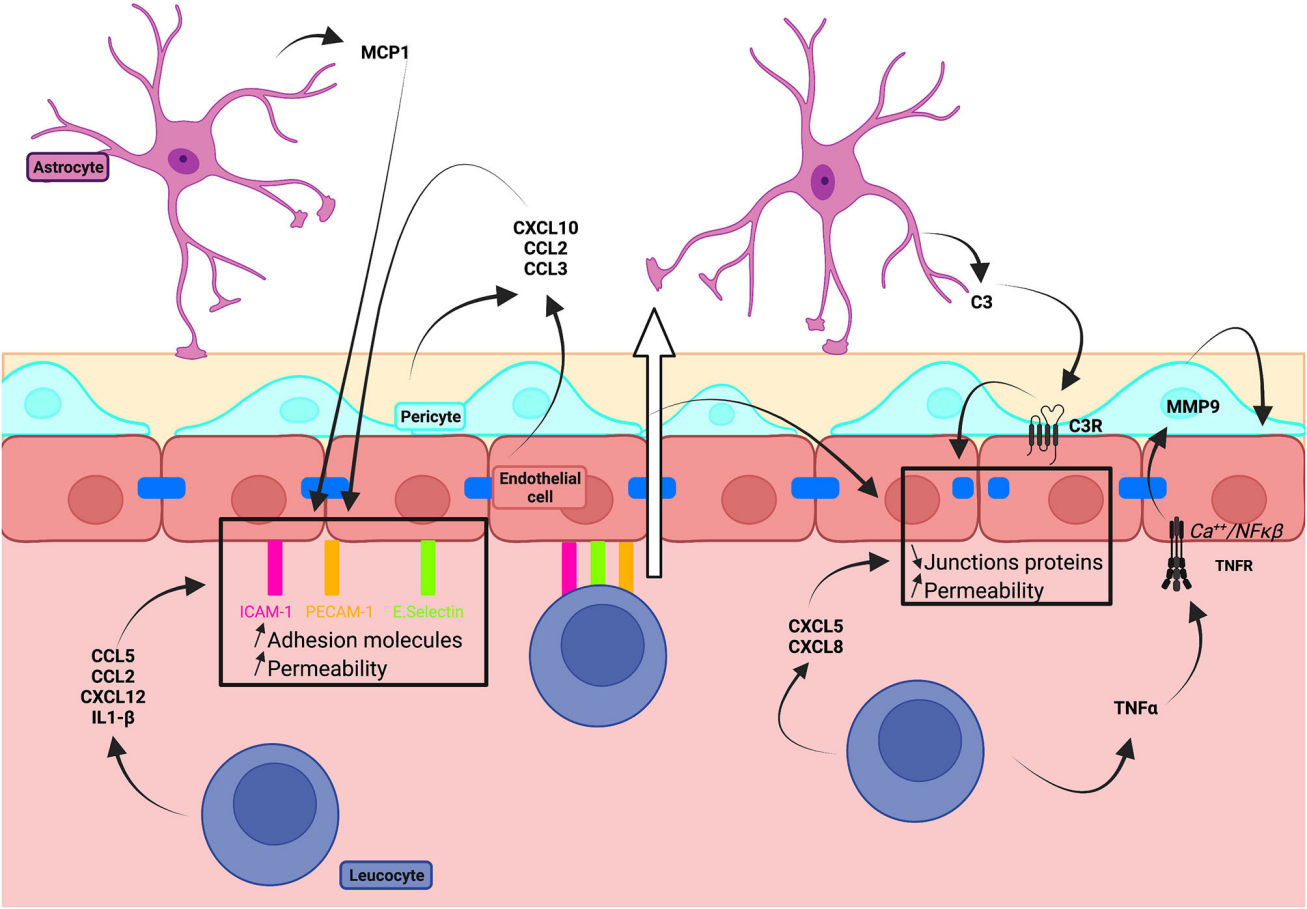
Schematic illustration of the impact of inflammatory processes on BBB integrity.

We review how BBB models have been used to examine the trafficking of peripheral immune cells across the barrier and the actions of these cells and molecules they secrete on BBB integrity (Figure 3).

### Immune Cell Trafficking Across the BBB

Transmigration of immune cells into the brain is crucial in pathologies with an inflammatory component, such as multiple sclerosis (MS). *In vitro* BBB models have revealed the role of BBB adhesion molecules, including ICAM-1, E-selectin, and PECAM-1, in transmigration of monocytes (Séguin et al., 2003), dendritic cells (Sagar et al., 2012), neutrophils (Wong et al., 2007), and lymphocytes (Wong et al., 1999). Subtypes of lymphocytes have differing migration properties with TH2 cells crossing the BBB most effectively (Prat et al., 2005). VLA-4 or integrin a4b1 binds VCAM-1 leading to the rolling of T cells and has been targeted in multiple sclerosis (Engelhardt and Kappos, 2008). In models based on HBMECs, the integrin a4 is expressed by endothelial cells. Recognition of integrin a4 by the soluble VCAM-1(sVCAM-1), which is present in the blood during inflammatory states, leads to TJ disruption (Haarmann et al., 2015) Work on BBB models using endothelial cells derived from CD34+ cord blood stem cells has shown that Th1 cells have enhanced migratory properties (Nishihara et al., 2021). Neutrophil transmigration reduced TEER (Wong et al., 2007) and monocyte migration was linked to the rapid remodeling of tight junctions expressing claudin-5 (Winger et al., 2014). In contrast, T lymphocytes did not affect the properties of HBMECs (Wong et al., 1999). Transmigration of dendritic cells has been associated with the restructuring of endothelial cell actin in a transwell model of hCMEC/D3 cultured with astrocytes (Meena et al., 2021).

Blood–brain barrier transwell models have been extensively used in studies on transmigration in different neuropathologies. Work on immune cells isolated from patients with multiple sclerosis revealed preferential migration of CD8+ and TH17+ T lymphocytes (Tsukada et al., 1993; Kebir et al., 2009). Interaction of the programmed cell death 1 molecule (PD-1) expressed by T cells with its ligands PD-L1 and PD-L2, which are expressed by isolated hBECs, was shown to be a key factor in migration (Pittet et al., 2011), whereas PECAM-1 was not crucial (Wimmer et al., 2019). In neurodegenerative diseases, the chemokines CXCL4 and CXCL10 facilitated the entry of lymphocytes derived from patients with Alzheimer's disease in cultures of hCMEC/D3 cells (Verite et al., 2017).

Transmigration of immune cells has also been studied in microfluidic BBB models. Endothelial cells and astrocytes have been cultured with hollow microfluidic fibers equipped with 4-μm pores to permit monocyte migration (Cucullo et al., 2002). A human model to study transmigration incorporated cultured endothelial cells, astrocytes, and shear stress in a 3D flow chamber device (Takeshita et al., 2014). A microfluidic system with transparent porous silicon nitride membranes designed to permit live imaging of T-cell transmigration has been developed with co-culture of brain endothelial cells derived from the human stem cells and bovine pericytes (Mossu et al., 2019). New generation models should enhance the understanding of transmigration.

### Immune Component Impact on BBB Integrity

In inflammatory states, endothelial cell expression of TJs proteins decreases and adhesion molecule expression increases (De Laere et al., 2017). Human models have been critical in the studies of the effects on the BBB of components of immune signaling, including chemokines, cytokines, and interleukins, secreted by peripheral cells during inflammation. BBB permeability has been shown to be decreased by serum from patients with MS applied to hCMEC/D3 cells (Curtaz et al., 2020; Sheikh et al., 2020) or by serum from patients with breast cancer and brain metastasis tested on CD34^+^-derived endothelial cells (Curtaz et al., 2020; Sheikh et al., 2020). These effects are mediated by downregulation of VE-cadherin, occludin, and claudin-5 and increased levels of oxidative stress in endothelial cell monolayers and co-cultured with pericytes. The MS serum contained high levels of proinflammatory interleukins, such as IL-17 and IL-26. These interleukins downregulated tight junction proteins in hCMEC/D3 monolayer or primary BECs (Li et al., 2017, 17; Setiadi et al., 2019; Broux et al., 2020).

*In vitro* BBB models have been crucial to establish links between inflammatory immune components and BBB degradation. In co-cultures of hCMEC/D3 with astrocytes, the chemokines, such as CXCL5 and CXCL8, transiently activated the Akt pathway, leading to ZO-1 redistribution and the appearance of actin fiber stress (Haarmann et al., 2019). TNF-α stimulation-induced oxidative stress and downregulated claudin-5 and occludin expression in HBMEC co-cultured with astrocytes under ischemic conditions (Abdullah et al., 2015). TNF-α also activated the Ca^2+^/NFκβ pathway in hCMEC/D3 cells inducing synthesis of the metalloproteinase MMP9, correlated with BBB degradation in several pathologies (Ding et al., 2019). Elements of the complement pathway were also involved in BBB damage. In co-culture models, astrocytic secretion of C3 activated endothelial cell C3a receptors which led *via* Ca^2+^-dependent signals, to decreased expression of TJs proteins and increased permeability (Propson et al., 2021).

Immune signaling components also enhance expression of adhesion molecules which assist immune cell extravasation. Chemokines linked to transmigration include CCL5 (Ubogu et al., 2006), CCL2 for dendritic cells (Sagar et al., 2012), or CXCL12 for T cells and monocytes (Man et al., 2012). The interleukin IL1β was shown to facilitate extravasation by delocalizing endothelial cell expression of the adhesion molecule integrin α5β1 (Labus et al., 2018). Astrocytes have been associated with secretion of MCP-1 (or CCL2), which assists monocyte migration by increasing ICAM1 and E-selectin expression in BECs (Weiss et al., 1998). Monocyte transmigration is facilitated by CXCL10, CCL2, or CCL3, which is secreted by pericytes and also hBECs (Chui and Dorovini-Zis, 2010; Niu et al., 2019).

Microvesicles released by immune cells may be another significant pathway for the liberation of immune signaling molecules. Microvesicles secreted by polymorphonuclear neutrophils decreased TEER in hCMEC/D3 models (Ajikumar et al., 2019).

## Neuronal Dysregulation and BBB Integrity

**Figure 4:**
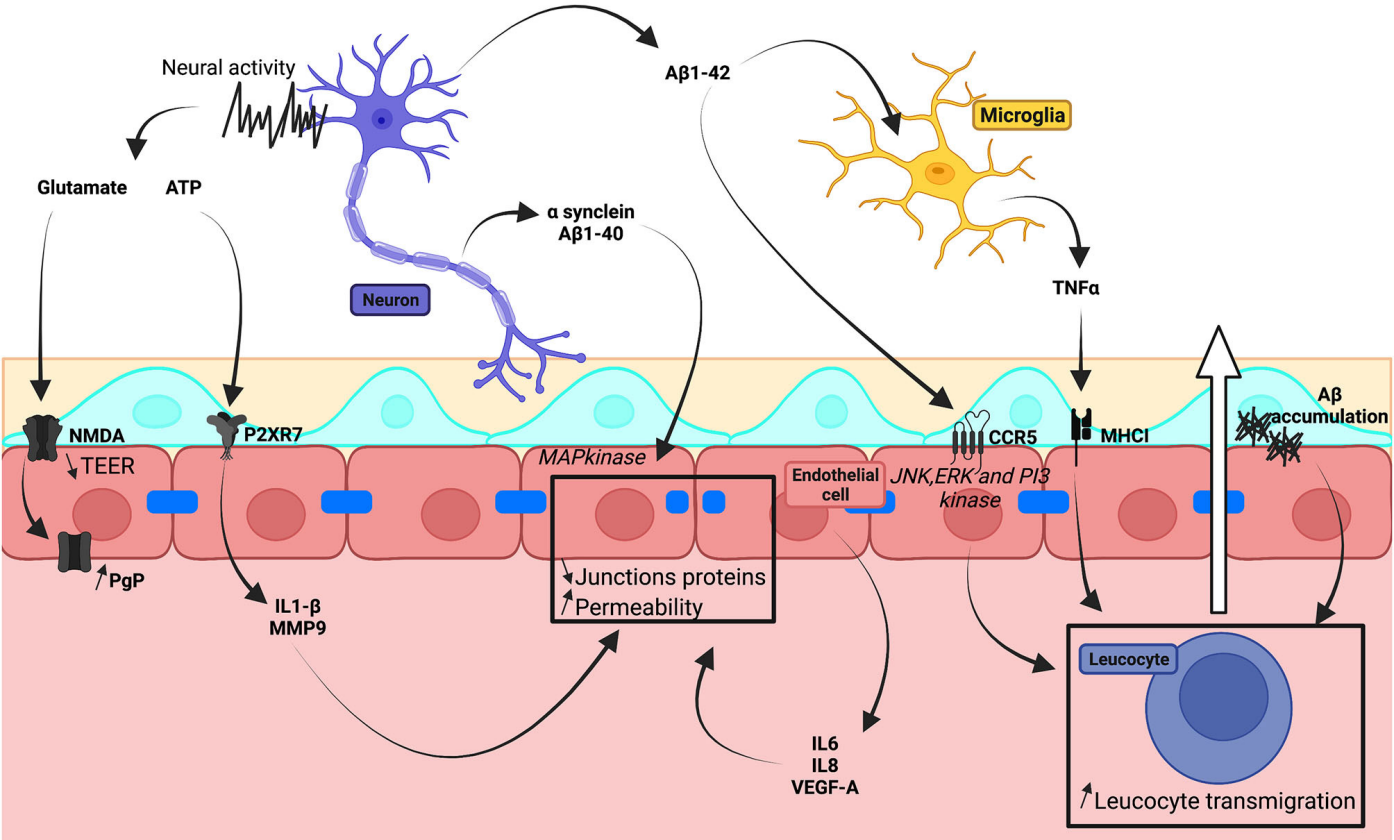
Schematic illustration of neural dysregulation on *in vitro* human BBB models.

Molecules linked to dysregulated neuronal processes contribute to BBB degradation (Figure 4). In Parkinson's disease, α-synuclein accumulation is linked to the loss of dopaminergic neurons. Neuronal damage in Alzheimer's disease is associated with amyloid-β (Aβ) plaques and tau-containing neurofibrillary tangles. Recombinant α-synuclein and Aβ1-40 activated the MAPkinase pathway in cells of hCMEC/D3 monolayers, so reducing expression of TJ proteins (Tai et al., 2010; Kuan et al., 2016). Fibrillar aggregations of amyloid-β increased BBB permeability (Parodi-Rullán et al., 2020). Application of amyloid-β1-42 to HBMECs upregulated CCR5, involved in T-cell transmigration, *via* JNK, ERK, and PI3 kinase pathways (Li et al., 2009). TNF-α, which is secreted by microglia stimulated by amyloid-β1-42, induced an increase in MHCI expressed by hCMEC/D3 and facilitated T-cell transmigration (Yang et al., 2013). The presence of amyloid-β1-40 or amyloid-β1-42 enhanced IgG uptake in iPSC-derived endothelial cells, differentiated using Qian's method, and stimulated inflammatory cytokine secretion (Mantle et al., 2016).

Neuronal activity may also release transmitter molecules, such as purines and glutamate, which might contribute to BBB degradation even if these effects have not yet been sufficiently studied. The hCMEC/D3 cell line expresses several purinergic receptors and also CD73 which converts AMP to adenosine (Mills et al., 2011). Activation of the P2XR7 receptor of hCMEC/D3 cells by ATP induced IL1β secretion, activated MMP9, and decreased ZO-1 and occludin expression (Yang et al., 2016). Glutamate decreased TEER in cultured monolayers of BEC lines although NMDA increased the same parameter (Neuhaus et al., 2011). In co-cultures of BECs and astrocytes derived from iPSCs of patients with familial SOD1 ALS, glutamate increased expression of the transporter P-gp *via* NMDA receptors. This effect was absent in cells derived *via* iPSCs of patients with the C9orf42 mutation, differentiated in a chemically-defined medium (Mohamed et al., 2019).

## Impact of Brain Invasion on BBB Integrity

**Figure 5:**
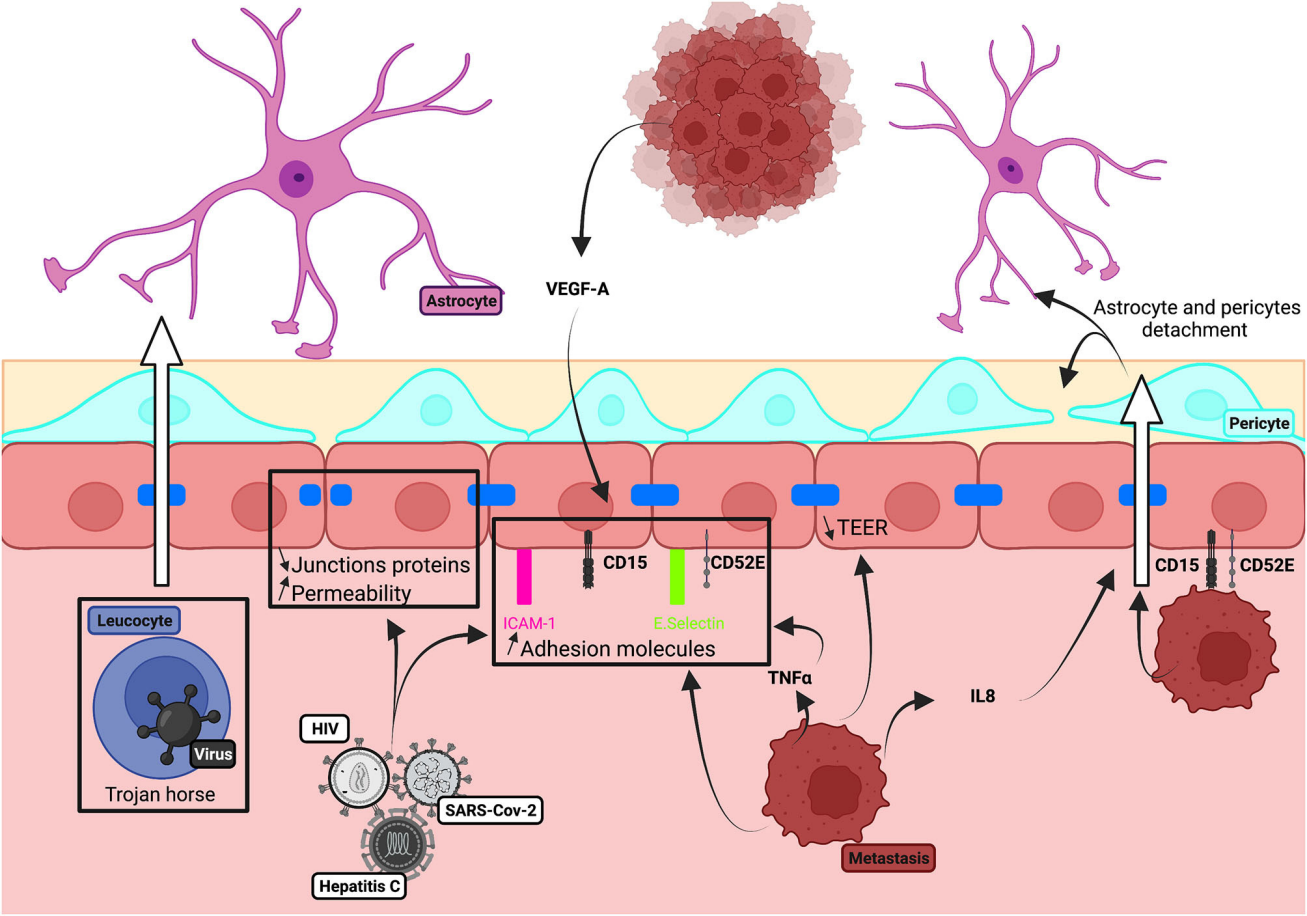
Schematic illustration of brain invasion on *in vitro* human BBB models.

We explore how neurotrophic viruses and metastatic cancer cells enter the brain *via* the BBB (Figure 5).

### Neurotrophic Virus

The BBB is a major route for neurotropic viruses to enter the brain where they may induce encephalitis, epileptic seizures, or stroke (Ludlow et al., 2016). Viruses, including human immunodeficiency virus (HIV-1), hepatitis C, and severe acute respiratory syndrome coronavirus-2 (SARS-CoV-2), reduce TJ integrity and induce the expression of adhesion molecules and cytokines (Liu et al., 2009; Strazza et al., 2011). These actions have been studied in BBB models. The Zika virus (ZIKV) is a mosquito-borne flavivirus, which causes microencephaly in the newborn and is linked to adult disorders, including encephalitis (Carod-Artal, 2018). Infection of primary human BECs with ZIKV decreased TJs expression, most severely for the Honduras-ZIKV strain. The micro-RNA, hsa-miR-101-3p, disrupted AJ and TJ proteins after ZIKV infection of hBMVEC cells and hCMEC/D3 (Bhardwaj and Singh, 2021). Interestingly, TJ degradation did not affect BBB permeability in work on BMECs (Leda et al., 2019), iPSC-derived endothelial cells differentiated from Qian's method (Alimonti et al., 2018), or on HUVEC cells (Clé et al., 2020).

Human immunodeficiency virus−1 causes neurological disorders, including cognitive decline and dementia in 40–60% of patients, even with tri-therapy (Anthony et al., 2005). BBB disruption during primary infection with HIV-1 (Rahimy et al., 2017) has been linked to neurological disorders (Chaganti et al., 2019). HIV-1 disrupted claudin-5 (András and Toborek, 2011). A better understanding of interactions between HIV-1 and BBB cells is needed to develop new therapeutic approaches.

Severe acute respiratory syndrome coronavirus-2, the novel coronavirus which emerged in 2019, induces neurological symptoms (encephalitis, Guillain–Barre syndrome, epileptic seizures, ischemic stroke, and hemorrhagic stroke) as well as a severe acute respiratory syndrome. Viral mRNA and proteins penetrate Central Nervous System (CNS) and olfactory bulb endothelial cells (Davies et al., 2020; Solomon et al., 2020). Work with human BBB models to understand how the virus enters the brain has focused on interactions between the coronavirus spike protein and the angiotensin-converting enzyme 2 (ACE2) receptor expressed by BECs. Data from co-cultures of CD34+ blood-derived endothelial cells and bovine pericytes suggest that SARS-CoV-2 does not directly infect endothelial cells (Constant et al., 2021). While the coronavirus spike protein did not compromise endothelial cell viability, BBB permeability increased, ZO1 expression was altered, and inflammatory cytokines, metalloproteases, and integrins were induced in hBMVECs (Buzhdygan et al., 2020). Hypoxia has recently been shown to enhance ACE2 expression by hCMEC/D3 cells and probably increases susceptibility to infection (Imperio et al., 2021).

Diverse mechanisms have been proposed for neurotropic virus entry into the brain. They include infection of hBECs of the BBB, transcytosis, and also infection of transmigration-competent immune cells which may enter the brain as a “Trojan horse.” A transwell 3D co-culture of HBMECs with astrocytes and monocytes showed how ZIKV-infected monocytes could cross the BBB to enter brain tissue (Bramley et al., 2017). ZIKV induction of adhesion expression molecules by endothelial cells facilitated the transmigration of infected monocytes in a transwell system using human CD34+ cells (Clé et al., 2020). The HIV-1 also enters the brain *via* infected monocytes (Nottet et al., 1996; Banks et al., 2001). BBB models have attempted to distinguish between endocytosis and Trojan mechanisms of entry. Lymphocytes infected with HIV-1 were shown to induce adhesion molecules which facilitated transmigration across HUVEC cells (Romero et al., 2000). The chemokine CCL2 mediates migration that increased BBB permeability, as shown when HIV-1 infected lymphocytes were added to transwells with astrocytes and BECs (Eugenin et al., 2006).

### Cancer Cells

Malignant glioma cells infiltrate peri-vascular spaces and displace astrocytic end-feet from BECs (Cuddapah et al., 2014; Watkins et al., 2014). However, the phenomenon may not be uniform since MRI scans in patients with glioblastoma have shown that the BBB is intact in some brain regions (Sarkaria et al., 2018). Most studies on BBB modulation in gliomas have focused on the delivery of compounds to alleviate symptoms. The important role of BBB in pathogenicity and morbidity is confirmed by the increases in permeability detected in high-grade, fast-growing, but not low-grade tumors.

Brain tumors could result from the metastatic spread of breast, lung, and melanoma cancers *via* blood vessels or nerves. The key role of claudin-5 in controlling hCMEC/D3 cell permeability and metastatic cancer cell migration has been established in protein silencing or overexpression studies on hCMEC/D3 cell lines (Ma et al., 2017). Furthermore, glioma cells secrete exosomes, enriched in the growth factor VEGF-A, which also reduces the expression of claudin-5 and occludin by endothelial cells *in vitro* (Yang et al., 2016).

Metastasis was enhanced in inflammatory conditions when HBMECs were co-cultured with lung cancer cells. The proinflammatory cytokine TNF-α enhanced the adhesion of metastatic cells to BECs, by increasing BEC expression of adhesion molecules and increasing the expression of their ligands by cancer cells (Wang et al., 2021). TNF-α increased the expression of CD15 and E-selectin adhesion molecules by hCMEC/D3 monolayers. CD15 and CD52E underly metastatic adhesion to brain endothelial cells (Jassam et al., 2017). Extravasation of metastatic cells has been studied in a 3D microfluidic platform in which human iPSC-derived BECs from Lippman's method supplemented by VEGF, astrocytes, and pericytes were exposed to breast tumor cancer cells. This work underlined the implication of CCL2/CCR2 axis for metastatic cell extravasation. Furthermore, serum from patients with breast cancer has been shown to increase BBB permeability in transwell co-cultures of endothelial cells (Hajal et al., 2021).

Co-cultures have helped to establish the nature of interactions between glioma cells and BECs. Glioma cells decreased TEER and increased paracellular fluxes when co-cultured with hCEMC/D3 cells (Mendes et al., 2015). The spheroidal 3D structure of gliomas may be significant. Coculturing glioblastoma spheroids with GBM spheroid and hCEMC/D3 cells has revealed a specific role of IL-8 in promoting tumor growth and tumor migration (McCoy et al., 2019). These data show how BBB models have an advanced understanding of mechanisms of tumor cell invasion and their effects on brain vasculature.

## Impact of Cerebrovascular Disease on BBB Integrity

**Figure 6:**
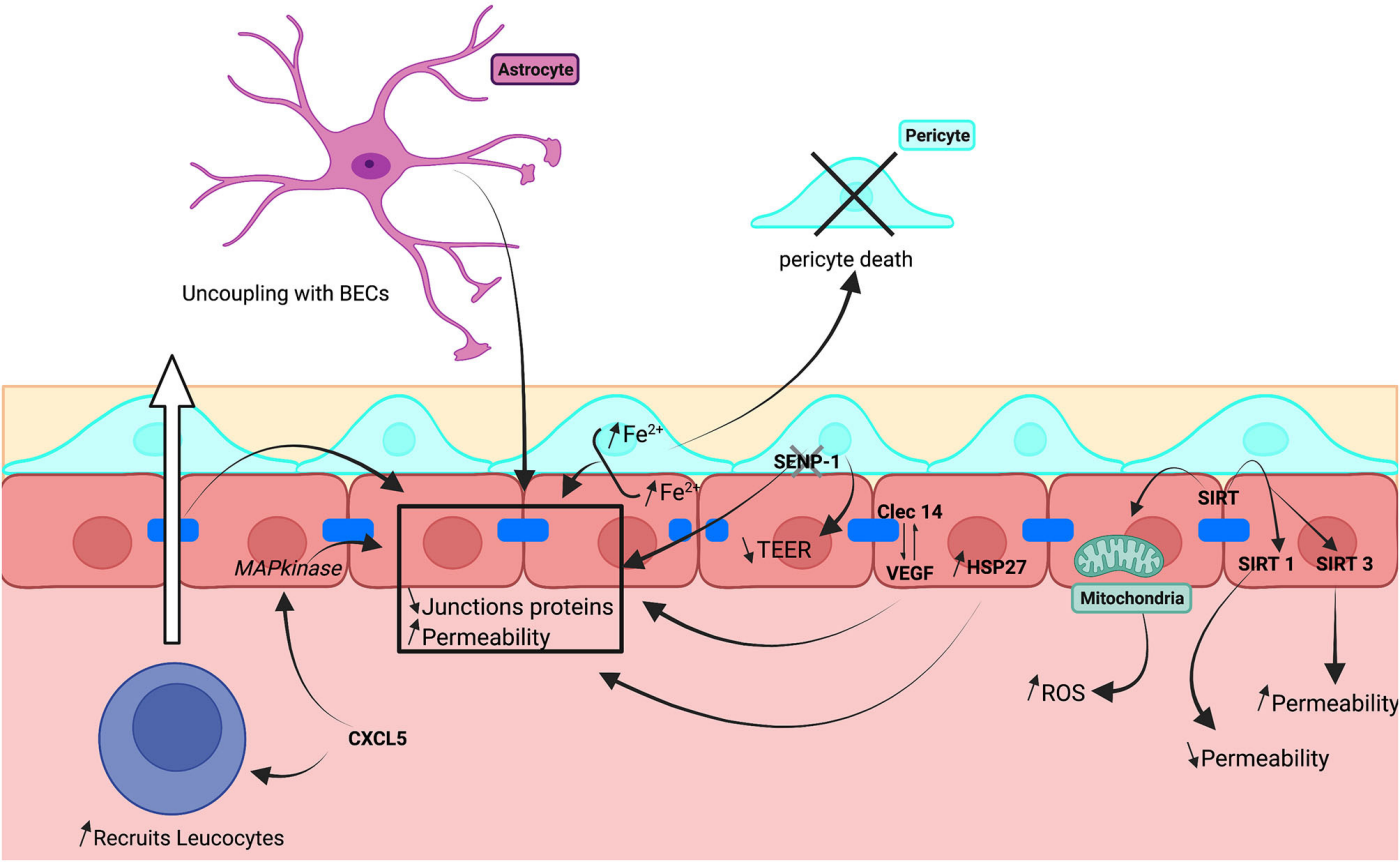
Schematic illustration of cerebrovascular dysregulation on *in vitro* human BBB models.

Cerebrovascular syndromes, including stroke, vascular malformations, vascular dementia, and edema related to post-traumatic epilepsy, are closely linked to BBB dysfunction (Andjelkovic et al., 2020). These conditions are associated with a hyperpermeability of the barrier, cellular cytotoxicity, and inflammation (Figure 6).

Cerebral amyloid angiopathy (CAA) is a cerebrovascular disease, often associated with Alzheimer's dementia, which results from amyloid-β accumulation on small blood vessel walls and often causes cerebral hemorrhage (Puy and Cordonnier, 2019). Blood vessels degenerate and inflammation linked to the vascular adhesion protein 1 and semicarbazide-sensitive amine oxidase (VAP1/SSAO) emerges. In BBB model cultures consisting of endothelial cells, neurons, and astrocytes, VAP1/SSAO induced hBEC secretion of IL-6, IL-8, and VEGF-A and decreased expression of the tight junction proteins ZO1 and claudin-5 (Solé et al., 2019).

Oxygen and glucose deprivation (OGD) mimics ischemic stroke-induced hyper-permeability of HBMECs due to internalization of VE-cadherin and is dependent on activation of the RhoA/Rock pathway (Chen et al., 2019). Pericytes and astrocytes are important in the BBB response to ischemia. A co-culture model with pericytes and astrocytes as well as endothelial cells showed that OGD increased intracellular levels of Fe^2+^ in BECs and pericytes and was correlated with the loss of TJs, AJs, increased BBB permeability, and pericyte death (Imai et al., 2019). Deletion of the SUMO-specific protease SENP-1 in pericytes of HBMEC/pericyte co-cultures revealed a protective role for this enzyme during ischemia. In the absence of SENP-1, TEER was reduced and decreased ZO-1 and occludin expression by BECs was decreased (Sun et al., 2020). Co-cultures of hCMEC/D3 cells with astrocytes show that both ischemia (Gerhartl et al., 2020) and traumatic injury (Augustine et al., 2014) induce an anatomical uncoupling of the two cell types mediated by the activation of MAPKinase pathways. Recovery after trauma, including a stabilized BBB permeability, reduced inflammatory processes and re-establishment of TJs was partly dependent on the hedgehog pathway in astrocytes (Wu et al., 2020).

Several proteins have been linked to the hyperpermeability of hBECs. One is the heat shock protein 27 (HSP27), which inhibits actin polymerization. Overexpression of HSP27 in endothelial cells reduced TJ protein expression and BBB permeability due to OGD treatment (Shi et al., 2017). Another protein, the C-type lectin domain containing 14A (Clec14A), which interacts with VEGF and is involved in angiogenesis, has been associated with the loss of TJs during ischemia (Kim et al., 2020). Sirtuine 1 & 3 (SIRT1 & SIRT3) proteins are involved in resistance to cellular stress, but have opposing effects in human BBB models. SIRT1 is protective and decreased BBB permeability, whereas SIRT3 increased permeability. SIRT effects have been associated with reactive oxidative stress generated by mitochondria (Chen et al., 2018).

Ischemia initiates massive inflammatory processes. CXCL5, a secreted chemokine that recruits neutrophils, has been linked to a reduced TEER and ZO-1 expression mediated *via* activation of p38 MAPkinase pathways in primary BECs (Yu et al., 2021). OGD treatment to mimic ischemic stroke downregulated the micro-RNA let-7 in hBEMCs. Overexpression protected against inflammatory processes inducing decreased TLR4 and MMP9 expression as well as reducing inducible nitric oxide synthase responses to OGD stimulation (Xiang et al., 2017).

The most frequent vascular formation syndrome is cerebral cavernous malformation. This syndrome results from mutations in CCM genes and is characterized by vascular dilatations. Deleting CCM3 protein in monolayers of human endothelial cells induced internalization of TJs proteins and diminished interactions with the actin cytoskeleton (Stamatovic et al., 2015).

*In vitro* human BBB models have been also used to test potential treatments. Therapy using progenitor endothelial cells may promote brain repair and angiogenesis after ischemia. The secretome of endothelial progenitor cells is suggested to promote angiogenesis and BBB recovery after OGD treatment in a co-culture model of BECs and pericytes. Recovery was characterized by upregulation of junctional proteins and expression of ICAM1 and VCAM1 (Loiola et al., 2021).

## Discussion/Conclusion

The progression of multiple brain pathologies involves BBB disruption. Therapeutic measures to alleviate this influence might be developed from work on humans, on animal models or as we argue here on models of the BBB derived from human cells. Human studies of BBB are challenging and have mostly focused on permeability measurements. The best insights into BBB permeability in human have been obtained from data on albumin concentrations in CSF and in the blood. While this technique is too invasive to be used frequently, alternatives involving non-invasive imaging, such as positron emission tomography (PET) or dynamic contrast-enhanced magnetic resonance imaging (DCE-MRI), could be useful. *In vivo* animal models remain close to the BBB architecture. However, there are significant molecular differences between mice and humans in tight junction proteins and some transporters (Urich et al., 2012; Hoshi et al., 2013; Song et al., 2020). The group of Weiss-Coray has recently published data on differences in gene expression between patients with AD and mouse models of AD (Yang et al., 2022). These molecular differences may partly explain the poor translation of promising molecules from animal experiments to successful clinical trials. *In vitro* human models of the BBB therefore seem to be the technique of choice for further studies.

This review has described an intense activity resulting in numerous publications on *in vitro* human BBB models. Creating a physiological cerebrovasculature *in vitro* and understanding its disruption in different pathologies have been a major challenge. We feel that the present is an appropriate moment to standardize protocols. We have listed at least 4 different origins of human endothelial cells and 5 types of 2D or 3D models. Differences also exist in cell supports and culture media for the same cell line. Validation protocols could also be standardized permitting comparison between models and decisions on the most appropriate BBB model to study specific neuropathologies. Our review has shown that novel mechanisms have been elucidated from both relatively simple transwell monolayers of endothelial cells as well as technically more complex, and more expensive, 3D BBB-on-chip models.

Results derived from different models have sometimes proved controversial. Using primary BECs, Biernacki et al. (2001) revealed that TH2 T-cells have a better migration than TH1-T cells whereas using CD34+-derived BECs revealed similar migration for TH1 and TH2 cells while TH1 cells migrated more effectively under inflammatory conditions (Nishihara et al., 2021). Interestingly, a study comparing the migration of TH1 cell line across a monolayer of mouse cell line bEnd5 or primary brain mouse endothelial cells revealed a longer crawling of T cells in bEnd5 and a decrease of diapedesis in primary cells (Steiner et al., 2011). Eventually, TEER permeability values as well as price and technical complexity may be the key factors in deciding which model is most appropriate to a specific question.

Despite advances, including the use of pericytes and astrocytes in co-culture and simulation of the effects of shear stress in microfluidic models, we feel human BBB models could still be improved. Brain vasculature is not homogenous but is organized along an arteriovenous axis of arterioles, capillaries, and venules. Within this zonation, there are differences in cell types, shear stress, vessel diameter, and basement membrane which are crucial to the BEC phenotype. Transcriptomic analysis has revealed zonation-dependent molecular differences (Vanlandewijck et al., 2018), which may be important for next-generation *in vitro* human BBB models engineered to answer specific questions.

Present models largely ignore interactions of the BBB with significant elements of its environment. In the future, BBB models might consider to simulate interactions with the cerebrospinal fluid compartment and with the microbiota–gut–brain axis (MGBA). The blood–cerebrospinal barrier (BSCF) is localized in the choroid plexus and glymphatic system of lymphatic drainage. Dysfunction in CSF brain drainage has been associated with neuropathologies, including TBI and AD (Natale et al., 2021). Vascular dynamics regulate the glymphatic systems involved in CSF drainage. The BSCF, like the BBB, acts to protect the brain but is also a site of immune cells trafficking. Alcendor et al. (2013) have developed an organotypic model, including both CSF and CNS compartments coupled to the BBB. The microbiotic axis is clearly linked to the integrity of the BBB (Braniste et al., 2014) and is affected by neurodegenerative disease (Zhu et al., 2021). While mouse models have mostly been used to examine interactions between the MGBA and the BBB, this work could usefully be extended to *in vitro* human BBB models.

In conclusion, we suggest that *in vitro* human models of the BBB will be critical to further progress in understanding multiple brain pathologies. We hope that this review has clarified how a balance can be achieved among complexity, realistic physiology, and ease of use of different models. Validation and standardization will assist the choice of model for different questions. Facilitating access to realistic, high-performance systems (Boeri et al., 2021) seems likely to improve diagnostics, clinical applications, and understanding of different pathologies.

## Author Contributions

All authors listed have made a substantial, direct, and intellectual contribution to the work and approved it for publication.

## Funding

This work has been supported by ANR-21-CE14-0005-01- RPV21052DDA.

## Conflict of Interest

The authors declare that the research was conducted in the absence of any commercial or financial relationships that could be construed as a potential conflict of interest.

## Publisher's Note

All claims expressed in this article are solely those of the authors and do not necessarily represent those of their affiliated organizations, or those of the publisher, the editors and the reviewers. Any product that may be evaluated in this article, or claim that may be made by its manufacturer, is not guaranteed or endorsed by the publisher.

## Abbreviations

AQP4: Aquaporin-4
AD: Alzheimer's disease
PD: Parkinson's disease
HD: Huntington's disease
ALS: Amyotrophic lateral sclerosis
BBB: Blood–brain barrier
BECs: Brain endothelial cells
EC: Endothelial cells
ECM: Extracellular matrix
hBEC: Human brain endothelial cells
HBMEC: Human brain microvascular endothelial cells.
hCMEC/D3: Immortalized human microvessel endothelial cell line.
NVU: Neurovascular unit (endothelial cells, astrocytes, pericytes, ECM and neurons)
OGD: Oxygen and glucose deprivation (to mimic ischemia)
P-gp: P-glycoprotein
TEER: Transendothelial electrical resistance (TEER)
TJs: Tight junction proteins.
